# Role of flagella and type four pili in the co-migration of *Burkholderia terrae* BS001 with fungal hyphae through soil

**DOI:** 10.1038/s41598-017-02959-8

**Published:** 2017-06-07

**Authors:** Pu Yang, Miaozhi Zhang, Jan Dirk van Elsas

**Affiliations:** Microbial Ecology, Groningen Institute for Evolutionary Life Sciences, University of Groningen, Nijenborgh 7, 9747 AG Groningen, The Netherlands

## Abstract

*Burkholderia terrae* BS001 has previously been found to be able to disperse along with growing fungal hyphae in soil, with the type-3 secretion system having a supportive role in this movement. In this study, we focus on the role of two motility- and adherence-associated appendages, i.e. type-4 pili (T4P) and flagella. Electron microcopy and motility testing revealed that strain BS001 produces polar flagella and can swim on semi-solid R2A agar. Flagellum- and T4P-negative mutants were then constructed to examine the ecological roles of the respective systems. Both in liquid media and on swimming agar, the mutant strains showed similar fitness to the wild-type strain in mixed culture. The flagellar mutant had completely lost its flagella, as well as its swimming capacity. It also lost its co-migration ability with two soil-exploring fungi, *Lyophyllum* sp. strain Karsten and *Trichoderma asperellum* 302, in soil microcosms. In contrast, the T4P mutant showed reduced surface twitching motility, whereas its co-migration ability in competition with the wild-type strain was slightly reduced. We conclude that the co-migration of strain BS001 with fungal hyphae through soil is dependent on the presence of functional flagella conferring swimming motility, with the T4P system having a minor effect.

## Introduction

Due to a lack of connectivity of water-filled pores, or simply as a result of migration “barriers”, the soil environment is often not able to support the movement of bacterial cells over long distances^1^. However, bacteria can extend their living area with the help of soil fungi or related hypha-forming organisms, riding the so-called “fungal highway”^2^. For instance, *Pseudomonas putida* PpG7 gained the ability to cross air-filled pores in soil and spread in the presence of hyphae of the oomycete *Pythium ultimum* ^3^. Similarly, the soil saprotroph *Lyophyllum* sp. strain Karsten could mediate the dispersion of various bacterial strains through several soil types^4, 5^. More recently, studies using *in situ* tests showed that several bacterial groups can translocate through soil along with native fungi^6^. The movement to a new microhabitat enables such bacteria to utilize the locally-available nutrients, thus exploring and colonizing a novel niche^7^, for instance accessing degradable pollutants that occur in sites remote from the original microhabitat^3^. Moreover, biofilms formed by bacteria have been found around fungal hyphae^4, 8^, and there is evidence supporting the contention that these biofilms confer protection from adverse conditions to the fungal host^8^, to the benefit of host and bacterial associate alike^4, 9^.

Bacterial cells are able to move on solid surfaces in various ways, including swimming, swarming, twitching, gliding and sliding. Generally, swimming and swarming motility are aided by flagella, and twitching motility relies on type-4 pili (T4P)^10^. Flagella are macromolecular structures composed of three major substructures: (i) the basal body, which is embedded within the cell envelope as a platform. It stabilizes flagella, secretes the distal components and supplies the power for movement; (ii) the filament, which serves as the propeller, and (iii) the hook, which is a universal joint that connects the basal body to the filament^11–13^. More than 30 proteins are involved in flagellar biosynthesis, in a strict, complex and tiered transcriptional regulation network^10, 14, 15^. Compared to the flagellum, the T4P system is relatively simple; a core set of 12–15 proteins is essential for T4P assembly and function^11^. These proteins form a platform for assembly and a channel for pilin secretion. Several ATPases are involved that support assembly and disassembly of the pilus^16^. Besides motility, flagella and T4P have been reported to mediate bacterial cell adherence to surfaces^17, 18^.

In previous work, *B. terrae* BS001 was found to co-migrate through soil along with the hyphae of several fungi, including *Lyophyllum* sp. strain Karsten, *Trichoderma asperellum* 302 and *Fusarium oxysporum* Fo47^4, 8^. However, dispersal in soil without fungal hyphae was not detected^4^. Moreover, strain BS001 was always found to migrate in the canonical fungal growth direction and never in the opposite one, which was attributed to the older fungal mycelium becoming less active and changing the surface structure. Although motility was implied, no concrete evidence for the mechanism behind the fungal-assisted movement has been obtained so far. Moreover, although the type three secretion system (T3SS) was postulated to be involved in the interaction^4^, recent results obtained by us show that the T3SS merely enhances the movement in soil along with fungal hyphae, but is not essential^19^.

In the current study, we further explored the role of two motility- and adherence-associated cellular appendices, i.e. the flagellum and the T4P, in the migration of *B. terrae* BS001 along with growing fungal hyphae through soil. Previous analyses of the BS001 genome demonstrated the presence of sets of both flagellar and T4P biosynthesis genes^20^. Although these systems were not extensively studied, we here hypothesized that flagellar movement is essential for the co-migration ability of *B. terrae* and that type-4 pili might be involved as a ‘helper’ system at the fungal surface. Here, we show the results of experiments aimed at elucidating these roles, and included an analysis of the effect of pH as a potential driver of motility.

## Results

### Analysis of flagellar and type-4 pilus systems and construction of mutant strains

The *B. terrae* BS001 genome^20, 21^ was first examined for the presence of flagellar and T4P systems. By these analyses, we found the presence of one cluster of motility-related (flagellar and chemotaxis) genes, and several T4P-related genes. A total of 36 structural flagellar biosynthesis genes were located together on one contig (contig00012), encoding most basal body formation and hook formation proteins (Supplementary Table S1). Additionally, two flagellar transcription regulators, *flhC* and *flhD*, were found (contig00091), next to the chemotaxis-associated genes *cheDR*-*mcp*-*cheWAY* and two motor genes, *motA* and *motB* (Supplementary Table S1). According to the accepted model^22^, *B. terrae* BS001 harbours the genes for most of the structural proteins for flagellar biosynthesis, with only one missing (*flhA*, an export component). In addition, 13 T4P related genes were identified in the genome of BS001 (Supplementary Table S2). These genes are predicted to encode the platform assembly proteins PilM, PilN and PilO, the secretion pore PilQ, the pilus protein PilA and the peptidase PilD. The latter is predicted to cleave prepilin to produce mature pilin. Additionally, genes for the ATPases PilF and PilT were also found in the strain BS001 genome. PilF drives pilus extension, whereas PilT mediates its retraction^16, 23^. When compared to the T4P system found in *Synechocystis* sp. PCC 6083^24^, strain BS001 contains the genes for all proteins that are essential for T4P biosynthesis.

On the basis of the genomic information, we selected the *fliF* (flagellum) and *pilN* (T4P) genes to construct (knock-out) mutant strains. Both genes were found to occur in only one copy in the *B. terrae* BS001 genome. By using a double cross-over allelic exchange procedure, a 493-bp fragment was deleted from the *pilN* gene (Supplementary Fig. S1), and a 1704-bp fragment from the *fliF* gene (Supplementary Fig. S2). This yielded *ΔpilN* and *ΔfliF* mutant strains, respectively. The robustness of these mutants, that is, the precise excision of the exact DNA segments at the desired genomic locations, was shown by PCR-based approaches (Supplementary Fig. S1, Fig. S2). Additionally, the expression levels of flagellar genes adjacent to the mutated *fliF* gene were examined by qPCR of samples from the swimming agar, revealing no significant differences between the BS001 wild-type and BS001 *ΔfliF* mutant strains (Supplementary Fig. S3). These results demonstrated that no polar effect was found in the BS001 *ΔfliF* mutant strain.

### Fitness of the mutants

*B. terrae* BS001 wild-type was co-introduced with either BS001 *ΔfliF* or BS001 *ΔpilN* (1:1 ratio) into three broth media, i.e. LB and M9 supplemented with either glucose or glycerol (M9Glu and M9Gly), to assess the relative fitness of these strains under different medium conditions. The organisms grew faster in LB medium than in both supplemented M9 media, reaching stationary phase at around 24 h (Supplementary Fig. S4a, S4d). In M9Glu medium, it took 48 h to reach the stationary phase (Supplementary Fig. S4b, S4e), whereas it took 96 h to reach early stationary phase in M9Gly medium (Supplementary Fig. S4c, S4f). In contrast to the different growth rates, the proportions of mutant versus wild-type strains stayed stable, that is, close to the initial (1:1) levels, in all media along the different growth phases (BS001 *ΔpilN* versus the wild-type: P = 0.673 in LB, P = 0.540 in M9Glu, P = 0.073 in M9Gly. BS001 *ΔfliF* versus the wild-type: P = 0.855 in LB, P = 0.845 in M9Glu, P = 0.104 in M9Gly). Thus, the mutant strains did not show any fitness advantage or disadvantage versus the wild-type when grown in the three different media (P > 0.05). Also, the fitness of wild-type and mutant strains was examined in a similar set-up on swimming agar (Supplementary Fig. S5). Similar to the results described above, the organisms dispersed very well in the agar, reaching 75.0 ± 4.1 mm for the BS001 wild-type/*ΔpilN* mixture (Supplementary Fig. S5a) and 71.7 ± 6.2 mm for the BS001 wild-type/*ΔfliF* mixture (Supplementary Fig. S5c) at day 4. The cell densities reached 10^9^ cfu/g agar at day 4, both at the inoculation sites and migration front (Supplementary Fig. S5b, S5d). The proportion of both mutant strains in the total remained stable at the inoculation site (ANOVA, P = 0.701 for the *ΔpilN* /wild-type pair and P = 0.548 for the *ΔfliF*/wild-type one). For the former pair, no significant difference was found between the inoculation site and migration site (t-test, P = 0.840 at day 2 and P = 0.403 at day 4). In contrast, in the latter pair, no *ΔfliF* mutant was found at the migration front (Supplementary Fig. S5c).

### Presence of flagella in *B. terrae* BS001 wild-type and mutant strains and motility tests

**Figure 1:**
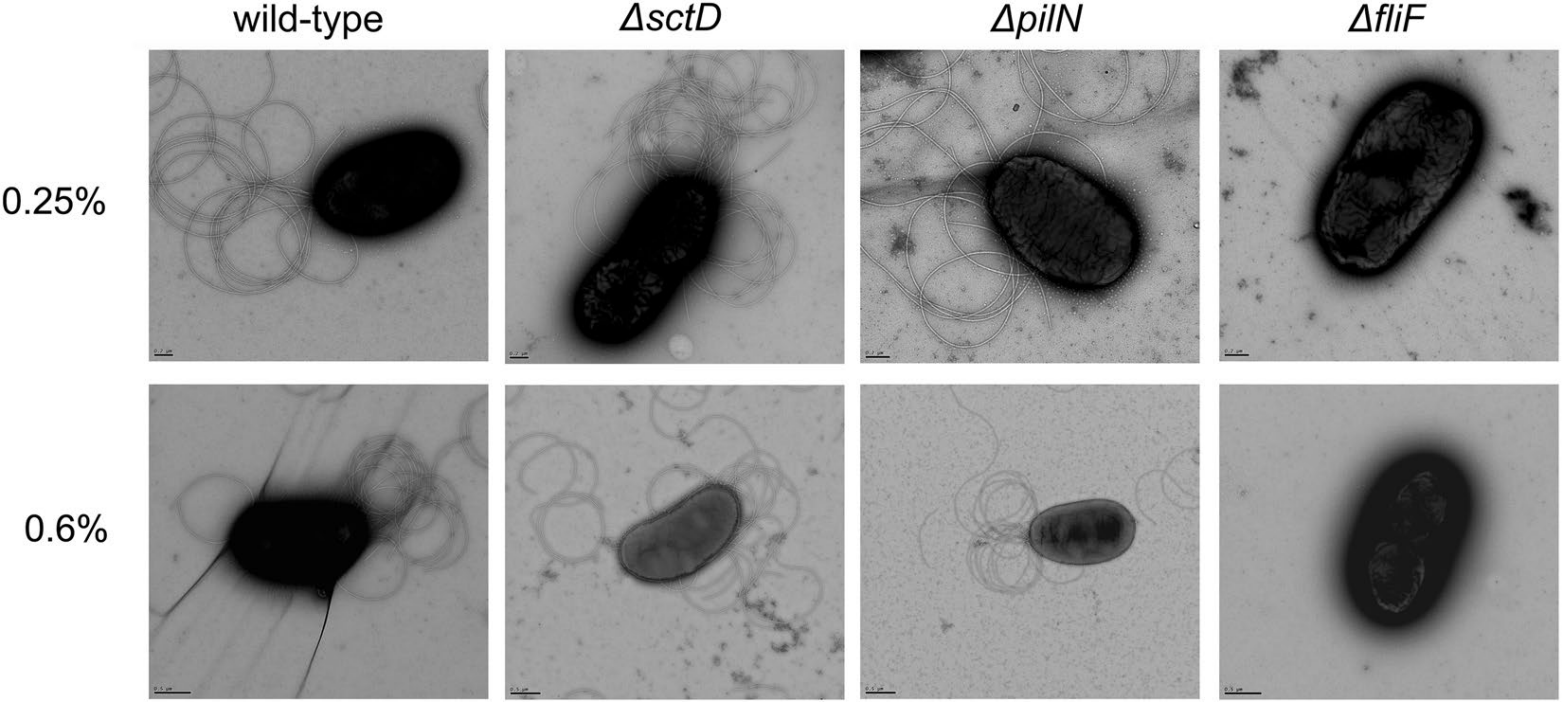
Electron microscopy images of *B. terrae* BS001 wild-type and mutant strains sampled from soft agar. 0.25%, sampled from 2.5 g/L agar; 0.6%, sampled from 6 g/L agar. Wild-type: wild-type BS001 strain; *ΔsctD*: BS001 *ΔsctD* mutant strain; *ΔpilN*: BS001 *ΔpilN* mutant strain; *ΔfliF*: BS001 *ΔfliF* mutant strain. All the flagellum-positive strains (wild-type strain, BS001 *ΔsctD* mutant strain, BS001 *ΔpilN* mutant strain) can synthesize flagella on semi-solid agar whereas BS001 *ΔfliF* mutant strain could not form any flagella.

Here, we studied the behavior of the two mutant strains versus the wild-type. We added a type three secretion system mutant strain described previously^19^, BS001 *ΔsctD*, as a flagellum-positive and cellular appendix-impaired control, in particular for the observation of flagella with transmission electron microscopy (TEM) and in the motility assay. Using TEM, all *B. terrae* strains presumed to have swimming capacity, i.e. *B. terrae* BS001 wild-type, BS001 *ΔsctD* and BS001 *ΔpilN*, were shown to possess bunches of polar flagella (Fig. 1). However, there were also ample free flagella in the medium, indicating the *B. terrae* BS001 flagella are very fragile and easy to break (Supplementary Fig. S6). In sharp contrast, we could not find any flagella in the TEM images of strain BS001 *ΔfliF* (cell-bound or free in the medium), indicating that the lack of a functional FliF protein abolished, to a very major extent, the capacity of strain BS001 to form functional flagella.

**Figure 2:**
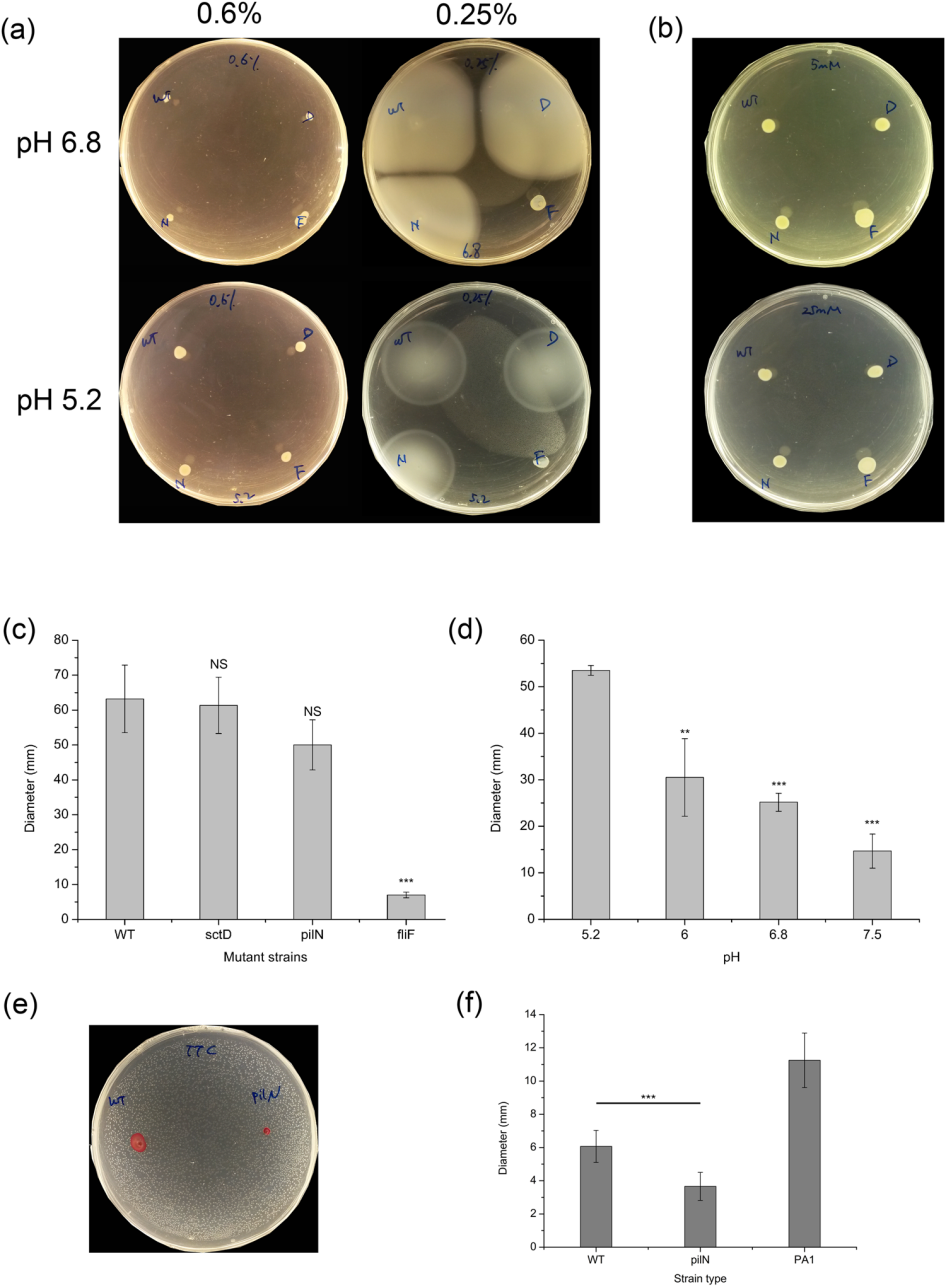
(**a**) Flagellar motility of *B. terrae* BS001 wild-type and mutant strains on soft agar medium. WT, wild-type strain; D, *ΔsctD* mutant strain; F, *ΔfliF* mutant strain; N, *ΔpilN* mutant strain. 0.25%, agar concentration 2.5 g/L; 0.6%, agar concentration 6 g/L. For swimming motility at pH 5.2, the image was obtained following overnight incubation, for the remainder, the pictures were taken 3 days after inoculation. (**b**) motility of *B. terrae* BS001 wild-type strain and mutant strains on swarming agar supplemented with 5 mM or 25 mM NaCl. (**c**) Swimming area (as described by diameter) of *B. terrae* BS001 wild-type and mutant strains on soft agar (2.5 g/L agar), 5 days after inoculation. (**d**) Swimming area of *B. terrae* BS001 wild-type strain on soft agar (2.5 g/L agar) at different pH, 24 h after inoculation. (**e**) Twitching motility of *B. terrae* BS001 wild-type and *ΔpilN* mutant strain (3 days after inoculation). (**f**) Twitching area of wild-type, *ΔpilN* mutant and *P. aeruginosa* PA1 (3 days after inoculation). *P < 0.05, **P < 0.01, ***P < 0.001 compared to wild-type in (**c**) and (**f**), compared to pH 5.2 in (**d**).

We then analyzed the flagellar motility (swimming and swarming) of all strains as related to pH, which is a modulator of flagellar motility due to proton-motive force. Considering swarming motility, we noticed that none of the four strains was able to move on the swarming agar (Fig. 2a), whereas the positive control produced swarming dendrites on the surface of the agar (data not shown). This in spite of the fact that the wild-type strain, the *ΔsctD* mutant strain and the *ΔpilN* mutant strain can all synthesize flagella (Fig. 1). Then, we analyzed the motor synthesis genes in the genome of strain BS001. The analysis showed that three pairs of *motA/motB* genes were present on the chromosome. MotA and MotB proteins have been reported to be powered by the proton-motive force and they may drive swimming as well as swarming motility^25^. We thus tested the potential swarming of *B. terrae* BS001 (wild-type and other strains) at lower pH, i.e. 5.2, and again found an absence of swarming. Finally, to understand the effect of a putative sodium motive force, 5 or 25 mM NaCl were supplied to the swarming agar (pH 6.8). In this experiment, swarming of the different flagellated BS001 strains also could not be detected (Fig. 2b).

With respect to swimming motility, all flagellum-positive strains, i.e. BS001 wild-type, BS001 *ΔsctD* and BS001 *ΔpilN*, were able to swim on the 2.5 g/L agar, at pH 6.8 as well as pH 5.2 (Fig. 2a). In contrast, the *ΔfliF* mutant strain had completely lost its swimming motility on this agar, as it did not spread to any extent on the agar surface (Fig. 2a), even after up to 5 days of incubation (data not shown). In the analyses on swimming agar at pH 6.8 (i.e. similar to the pH of the soil microcosm), the *ΔsctD* mutant strain (diameter of swimming area 61.3 ± 8.1 mm at day 5) did not show any delay of swimming compared to the wild-type strain (diameter of swimming area 63.2 ± 9.7 mm at day 5, P = 0.776, t-test). The *ΔpilN* mutant strain (diameter of swimming area 50.0 ± 7.2 mm at day 5) revealed a slightly but not significantly smaller dispersal front than the *ΔsctD* mutant (t-test, P = 0.144) as well as the wild-type strain (t-test, P = 0.070) (Fig. 2c). Additionally, the dispersal found for the wild-type strain BS001 at lower pH was greater than that observed at higher pH, as evidenced from Fig. 2d. The diameter of the swimming area reached 53.5 ± 1.1 mm at pH 5.2 after 24 h incubation, versus 30.5 ± 8.4 mm at pH 6.0 (t-test, P = 0.00113 compared to pH 5.2), 25.2 ± 1.9 mm at pH 6.8 (t-test, P < 0.001) and 14.7 ± 3.7 mm at pH 7.5 (t-test, P < 0.001). The latter values were all significantly different from the value at pH 5.2, indicating a clear pH effect on the degree of swimming.

Twitching motility was also examined for all strains, using a standard twitching motility test on 1% agar (a positive control is given in Figure S7). As shown in Fig. 2e, the BS001 wild-type strain expanded clearly at the surface between the agar and the Petri dish. However, the BS001 *ΔpilN* mutant strain showed significantly reduced motility [3.7 ± 0.8 versus 6.1 ± 1.0 mm (t-test, P < 0.001, Fig. 2f)].

### Influence of the T4P apparatus on the migration of *B. terrae* BS001 along with the hyphae of *Lyophyllum* sp strain Karsten and *T. asperellum* 302 in soil

**Figure 3:**
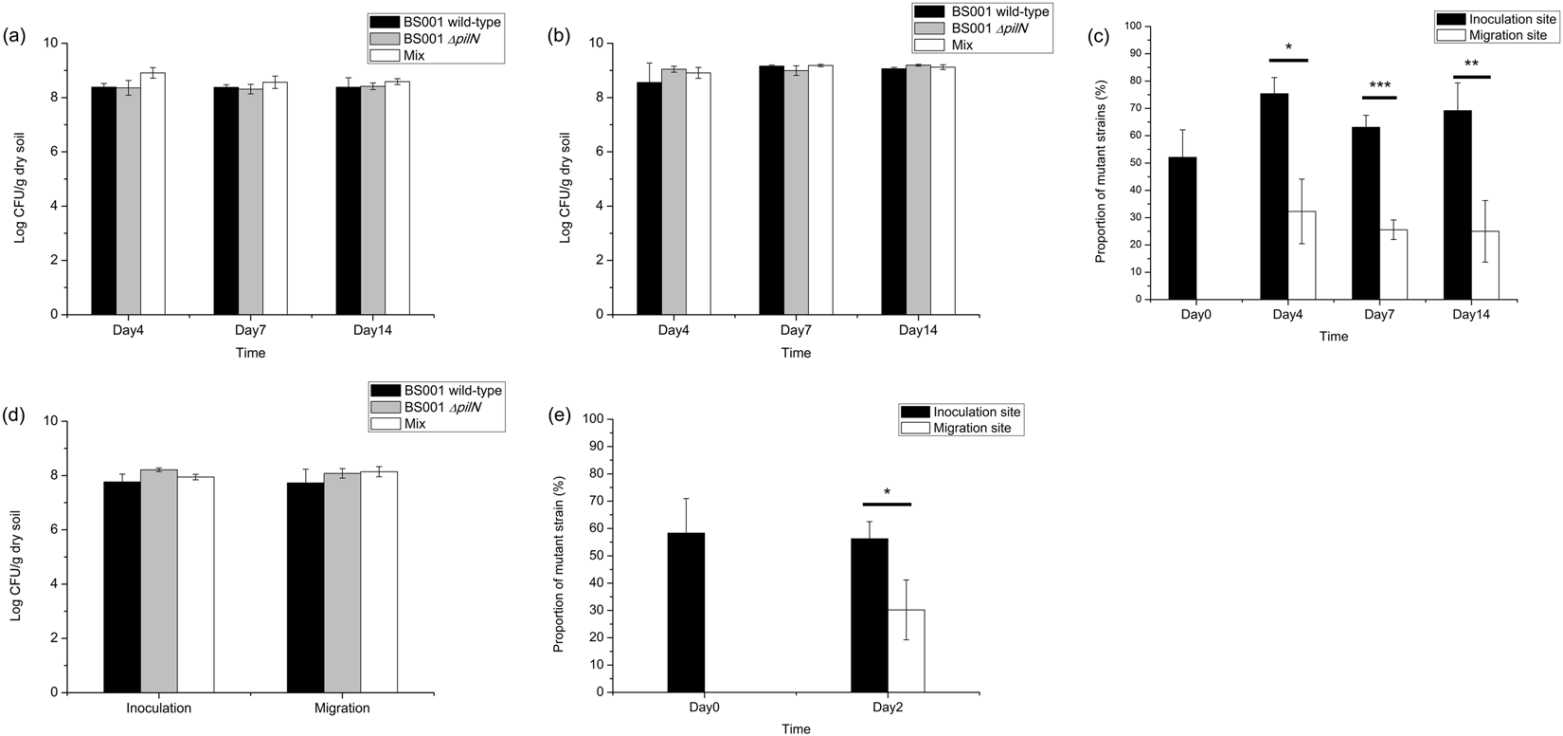
Population dynamics of BS001 wild-type and *ΔpilN* mutant strains in soil microcosms over time. (**a**) Cell density at the inoculation site, at *Lyophyllum* sp. strain Karsten. (**b**) Cell density at the migration site of *Lyophyllum* sp. strain Karsten. (**c**) Proportion of *B. terrae* BS001 *ΔpilN* mutant strain in the total BS001 population in the mycosphere of *Lyophyllum* sp. strain Karsten. (**d**) Cell density at day 2 in the microcosm of *T. asperellum* 302. (**e**) Proportion of *B. terrae* BS001 *ΔpilN* mutant strain in the total BS001 population in the mycosphere of *T. asperellum* 302. *P < 0.05, **P < 0.01, ***P < 0.001.

Both the wild-type and the *ΔpilN* mutant strain survived very well at the inoculation site with the emerging mycosphere of *Lyophyllum* sp. strain Karsten, reaching cell densities of around 10^8^ cells/g dry soil (Fig. 3a). Additionally, the BS001 wild-type and BS001 *ΔpilN* strains did show migration along with the growing fungal hyphae in soil microcosms, in separate systems (Fig. 3b), also reaching similar cell densities (up to 10^9^ CFU/g dry soil) at the migration front. Interestingly, when strain BS001 *ΔpilN* was introduced into the soil together with BS001 wild-type (1:1), the proportion of the mutant strain in the mix at the migration site decreased, from 52.1 ± 10.1% to 32.3 ± 11.8% (t-test, P = 0.092) at day 4 and further down to 25.6 ± 3.5% (t-test, P = 0.023) at day 7, being kept at this level afterwards (Fig. 3c). In contrast, the proportion of the mutant strain in the population increased significantly, from 52.1 ± 10.1% at day 0 to 75.4 ± 5.9% at day 4 (t-test, P = 0.041) at the inoculation site. After this initial increase, it fluctuated at the subsequent time points, i.e. 63.1 ± 4.4% (t-test, P = 0.180, compared to day 0) at day 7 and 69.2 ± 10.2% at day 14 (t-test, P = 0.107, compared to day 0, Fig. 3c). However, the relative abundances of the mutant strain at the migration fronts were significantly lower than those at the inoculation site at all sample time points (t-test, P = 0.011 at day4, p < 0.001 at day7, P = 0.007 at day 14). The actual CFU counts that underlie these percentages can be found in Table S3.

The behavior of strains BS001 and BS001 *ΔpilN* in the *Trichoderma asperellum* 302 mycosphere was similar to that in the mycosphere of *Lyophyllum* sp. strain Karsten when the two strains were introduced separately. Both strains survived well at the inoculation site (around 10^8^ CFU/g dry soil, Fig. 3d) and migrated along with the growing fungal hyphae, reaching about 10^8^ cells/g dry soil (Fig. 3d). However, when they were inoculated in a 1:1 mix, the proportion of BS001 *ΔpilN* at the inoculation site was 56.3 ± 6.3% at day 2, which was similar to the initial level, i.e. 58.3 ± 12.6% (t-test, P = 0.814) at day 0. In contrast, it decreased significantly, to only 30.2 ± 11.0% at the migration site at day 2 (t-test, P = 0.0375, Fig. 3e). The actual CFU counts that underlie these percentages can be found in Table S3.

### Influence of flagella on the migration of *B. terrae* BS001 along with the hyphae of *Lyophyllum* sp strain Karsten and *T. asperellum* 302 in soil

**Figure 4:**
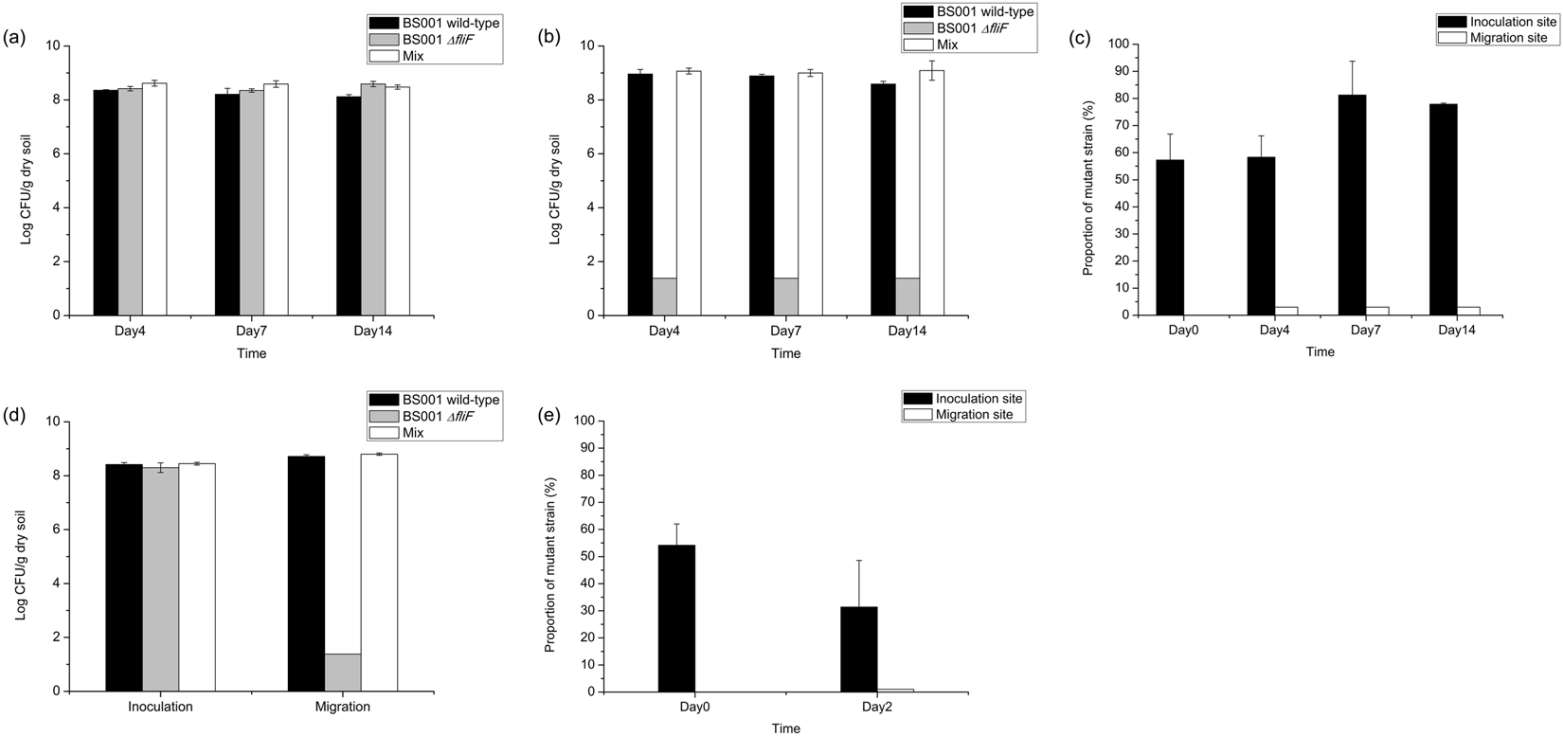
Population dynamics of wild-type and *ΔfliF* mutant strains in soil microcosms over time. (**a**) Cell density at the inoculation site, at *Lyophyllum* sp. strain Karsten. (**b**) Cell density at the migration site of *Lyophyllum* sp. strain Karsten. (**c**) Proportion of *B. terrae* BS001 *ΔfliF* mutant strain in the total BS001 population in the mycosphere of *Lyophyllum* sp. strain Karsten. (**d**) Cell density at day 2 in the microcosm of *T. asperellum* 302. (**e**) Proportion of *B. terrae* BS001 *ΔfliF* mutant strain in the total BS001 population in the mycosphere of *T. asperellum* 302.

In soil microcosm with *Lyophyllum* sp. strain Karsten, the loss of the functional *fliF* gene in strain BS001 *ΔfliF* resulted in a complete abolishment of migration along with the soil-exploring fungal hyphae. As shown in Fig. 4, the wild-type strain BS001 dispersed as expected with the growing fungal hyphae. However, in the case of BS001 *ΔfliF*, no cells were detected at the migration site (detection limit 24 CFU/g dry soil) in the separate inoculation experiment (Fig. 4b). We then tested the ability of strain BS001 *ΔfliF* cells to migrate along with the fungus when accompanied by moving BS001 wild-type cells using 1:1 cell mixtures. Remarkably, no, or, if any, very low numbers of mutant cells were taken along with the wild-type ones, as evidenced by the fact that the proportions of BS001 *ΔfliF* cells in the total populations at the migration site dropped, from 57.3 ± 9.6% at the onset of the experiment (day 0 at inoculation site), to below the detection limit in all measurements (Fig. 4c). However, strain BS001 *ΔfliF* did survive at the inoculation site (Fig. 4a), and even increased in abundance, reaching up to 10^8^ CFU/g dry soil from the 5 × 10^5^ introduced ones. Moreover, strain BS001 *ΔfliF* outcompeted the wild-type strain in the (1:1) mixed-inoculant treatment. The proportion of BS001 *ΔfliF* in the mixed population changed from 57.3 ± 9.6% at day 0, via 58.3 ± 7.9 at day 4 (t-test, P = 0.891) and 81.3 ± 12.5 at day 7 (t-test, P = 0.0577) to 77.9 ± 0.4% at day 14 (t-test, P = 0.0648, Fig. 4c). The actual CFU counts that underlie these percentages can be found in Table S4.

Finally, the strain BS001 *ΔfliF* mutant showed a behavioral response to *T. asperellum* 302 similar to the afore described one. Much like with *Lyophyllum* sp strain Karsten, strain BS001 *ΔfliF* neither moved along with the growing hyphae (Fig. 4d), nor received detectable ‘help’ from the strain BS001 wild-type cells (Fig. 4e). In contrast, the strain BS001 *ΔfliF* mutant cells survived very well at the inoculation site (10^8^ CFU/g dry soil, Fig. 4d). Unlike what was found in the soil microcosm with *Lyophyllum* sp. strain Karsten, the proportion of *ΔfliF* mutant cells in the mixed population remained statistically similar at the inoculation site of *T. asperellum* 302, with 54.2 ± 7.9% at day 0 and 31.4 ± 17.2% at day 2 (t-test, P = 0.128, Fig. 4e). The actual CFU counts that underlie these percentages can be found in Table S4.

## Discussion

The flagellum and the T4P are the most commonly studied motility devices in bacteria. Polar flagella are known to act as helical propellers, whereas the T4P is a so-called ‘linear’ actor^26^. Also, both systems can be involved in bacterial attachment to surfaces^17, 18^. Here, our primary objective was to investigate if any of the two systems, or both, are involved in the spreading behavior of *B. terrae* BS001 along with fungal hyphae through soil^4, 8^.

Regarding the T4P system, PilN is one of the key proteins in the type-4 apparatus. It binds to the PilM protein to form an inner membrane platform for T4P biosynthesis^27, 28^ and is indispensable for a vital T4P system. Given previous transcriptome analyses with strain BS001 in a fungal confrontational assay, in which *pilN* gene expression was indeed found^29^, we here constructed a *ΔpilN* mutant by deleting a 0.5 kb central part of the *pilN* gene. This part has previously been reported to be essential for the function of the protein^27, 30^. The subsequent analyses of colony expansion in a standard twitching motility assay revealed that the loss of *pilN* resulted in significantly reduced expansion. In concordance with the literature^31, 32^, we interpret this reduction to indicate impaired surface twitching motility.

Then, again using the wild-type strain, we knocked out a 1.7 kb part of the *fliF* gene, which was predicted to encode the FliF protein, expecting this mutation to destabilize the flagellar apparatus. The FliF protein-containing inner membrane ‘MS ring’ protein complex is required for FliG protein stability^33^ and thus plays an essential role in the early stage of flagellar assembly ^22, 34^. The loss of FliF thus likely results in a complete collapse of the flagellar structure. Here, we found clear evidence for such a flagellar collapse, given the absence of any detectable flagella from the transmission electron microscopy pictures obtained with the *ΔfliF* mutant. Moreover, no morphological differences were observed regarding the colonies formed on solid agar. Thus, the truncation of single genes in the two systems, leaving the remaining genes intact (Supplementary Figure S3), did not result in any polar effect or detectably affect growth rates or morphologies. Given the premises that PilN and FliF are essential for the formation of the T4P and flagellar systems, we surmised that functional T4P and flagellum structures were not formed even if the other proteins were expressed.

Considering the current information on motility systems in *Burkholderia* spp, swarming motility has been found in *B. pseudomallei* ^35^, *B. glumae* ^36^ and *B. cenocepacia* ^37^, whereas twitching motility so far was found in *B. thailandensis* ^38^. However, the knowledge with respect to swarming or twitching motility in *B. terrae* has hitherto remained extremely limited. Notwithstanding the presence of other cellular appendices, such as lateral flagella^10^, swimming motility is typically powered by polar flagella. In this study, all flagellum-positive strains of *B. terrae* BS001, i.e. the wild-type, *ΔsctD* and *ΔpilN* mutants, were shown to produce polar flagella. Moreover, they were able to successfully swim in 0.25% agar, confirming that their polar flagellar systems were indeed functional. Additionally, *B. terrae* BS001 (in this case, the wild-type strain) produced a migration fronts that extended progressively further with decreasing pH (Fig. 2d). Thus, at lower pH the motility-supported spread of strain BS001 is higher under the experimental conditions. This is consistent with a previous study, which showed enhanced swimming motility at lower pH in *Salmonella enterica* serovar Typhimurium SJW1103^39^. The proton-motive force presumably plays a role in this, spurring flagellar rotation. In contrast, swarming motility is apparently supported by hyper-flagellation, i.e. the biosynthesis of lateral flagellar systems^40^ or by polar flagella with shifting of the motor^10, 41^. Swarming is driven by the sodium-motive force in *Aeromonas hydrophila* or by the proton-motive force in another *Aeromonas hydrophila*, *Bacillus subtilis* and *Pseudomonas aeruginosa* ^25^. Analysis of the MotA and MotB proteins in strain BS001 provided an indication for the tenet that the motility system in this strain is powered by the proton-motive force. Thus, we attempted to establish optimal conditions for any putative swarming motility using lowered pH (5.2 instead of 6.8) or the presence of NaCl, but failed to induce swarming motility in strain BS001. Moreover, we did not find any genes associated with the formation of lateral flagella, nor an increase of the number of flagella from swimming agar to swarming agar by TEM (Fig. 1). In contrast, *Pseudomonas aeruginosa* employed alternative motors (MotCD) to support swarming motility^40, 42^. In strain BS001, we did not find any predicted proteins with homology to MotCD. The absence of lateral flagella, or of alternative motors might, be the reason for BS001's failure to move on swarming agar.

Soil is often not water-saturated, which limits the water-incited connectivity and hampers swimming or swarming motility based bacterial migration over longer distances^1, 3, 43^. At the same time, several studies have reported that the cells of varying bacterial species are able to disperse through unsaturated soil systems along with “fungal highways”^2, 4–6^. However, the exact mechanisms behind this behavior were thus far not well understood for *Burkholderia* spp.

In previous work, the T4P system has been reported to be involved in cellular twitching motility under conditions of water stress (reduced water availability), as well as in biofilm formation, attachment and virulence^17, 18, 44^. In the current study, we found that the strain BS001 Δ*pilN* mutant was impaired in colony expansion, which was interpreted as twitching inhibition. It could still swim on semi-solid agar, as well as through the soil via growing hyphae of *Lyophyllum* sp. strain Karsten and *T. asperellum* 302. Whereas the partial loss of *pilN* did not affect the fitness of strain BS001 in LB and M9 media, nor on swimming agar, the Δ*pilN* mutant was apparently at a slight advantage at the inoculation site in the mycosphere in soil microcosms. The disruption of *pilN*, abolishing the assembly of the T4P system as well as the secretion of pilin, may have exerted a positive effect on survival as compared to the wild-type. Although we cannot easily explain this, it might relate to the impaired twitching and/or adherence. A similar effect may also be at the basis of the finding that the proportion of strain BS001 Δ*pilN* in the mixed population was lowered at the migration site as compared to the inoculation site. Thus, PilN is hypothesized to have a relatively minor role, as an adherence and/or twitching device, in the co-migration behavior. This is reminiscent of the putative role of the T3SS, as recently described^19^. The T3SS has previously been reported to promote bacterial migration with fungal hyphae through soil by helping in the adherence to binding sites at the hyphal tip. We conclude that the T4P system might act as an ‘enhancer’ that allows bacterial cells to disperse better at the fungal surface, either by adherence or by twitching, giving the T4P endowed cells an ecological “migration” twist.

Flagellar motility facilitates bacterial dispersion on surfaces, but it is restricted to a narrow range of conditions of wetness, which is related to the (soil) water potential^45^. With respect to the role of flagella, it was recently suggested that flagellated bacteria cannot move on water-unsaturated surfaces (such as high-concentration agar media), and their only way of movement would be with the help of a mycelial network^43^. In contrast, Hover *et al*. recently indicated that flagella are not essential for *Serratia marcescens* to migrate along with the hyphae of several zygomycetous fungi^46^. Here, our collective data provide convincing evidence for the tenet that the presence of functional flagella is essential for strain BS001 cells to move along with fungal mycelia to remote locations in soil. The disruption of *fliF* resulted in the complete functional impairment of the flagellum, i.e. the loss of swimming motility (Fig. 2a and c) and, concomitantly, the loss of co-migration ability with fungal hyphae (Fig. 4b and d). Considering the fact that strain BS001 was indeed a swimmer, but not a swarmer (on soft agar plates), we surmised that its migration along with growing fungal hyphae in soil was associated with swimming motility. Additionally, if this migration with fungal hyphae would be dependent on swarming motility, which is often an orchestrated multicellular behavior, the *fliF* mutant strain might actually act as a “cheater”, being carried as cargo by the moving wild-type group in mixed culture^47^. However, no obvious helper effect was detected in such a mixed culture, down to the limit of detection (Fig. 4c and e). Thus, the migration of strain BS001 with extending fungal mycelia through soil depends on individual behavior (i.e. swimming) and is critically dependent on flagella that mediate swimming motility.

Although there is evidence obtained with organisms like *Pseudomonas putida* KT2440 that dispersal along with fungal (*Morchella crassipes)* hyphae is driven by flagellar motility^43^, we hitherto did not understand the mechanisms that *B. terrae* BS001 uses to co-migrate along with growing fungal hyphae. In previous work, Nazir *et al*.^48^ proposed a model that, in a loose manner, involved flagella-mediated and T3SS-supported bacterial motility and attachment. Recent work demonstrated that the T3SS may play a relatively minor (adherence-related) role in this process^19^. In the current study, we examined the role of T4P and flagella. Based on thedata obtained, we argue that *B. terrae* BS001 most likely moves along with fungal hyphae through soil on the basis of flagellar-driven swimming motility. Moreover, the T4P system, like the T3SS, appears to foster the migration process, most likely by offering to the cells a device for (ephemeral) anchoring of, or twitching-driven movement towards, the hyphal tip, where nutrients become available. Thus, we here provide data that refine the Nazir *et al*. model of *B. terrae* BS001 migration along with fungal hyphae in soil. The model predicts that bacterial cells can swim along with the growing fungal hyphae, with some (temporarily) attaching to the growing/extending hyphal tip, where materials abound that drive bacterial cell division. Then, some cells from the putative microcolony formed at the hyphal tip may get dislodged and swim out in the forward direction, thus (again) accompanying the extending hyphae. This adherence/growth/swimming cycle may thus repeat, accompanying the growing fungal hyphae.

In conclusion, our data demonstrate that the migration of *B. terrae* BS001 along with fungal hyphae that explore a soil habitat is critically dependent on the ability of the bacterium to swim. Flagella are essential for the motility on swimming agar, as well as in the co-migration of strain BS001 with the two selected soil-exploring fungi, as such behaviour was completely abolished in the absence of a functional flagellar system. Moreover, the T4P system was shown to promote the bacterial movement along with the extending hyphal tips, as a migration ‘enhancer’.

## Materials and Methods

### Strains and culture conditions

*Burkholderia terrae* BS001 wild-type strain^4^, and associated mutant strains were cultured in LB broth (Sigma-Aldrich Co., USA), with shaking, or on R2A agar (Difco, USA) at 28 °C. The construction of a T3SS mutant strain, *B. terrae* BS001 *ΔsctD*, was described in a previous study^19^. In order to construct a T4P mutant strain, the *pilN* gene was knocked out via a double crossover based allelic exchange using suicide vector pSUP202 (chloramphenicol resistance)^49^. Using the same strategy, the *fliF* gene was knocked out in order to produce a flagellum-negative mutant strain. For details about the construction of the mutant strains, see the electronic supplementary material, Supplementary Methods. The fungal hosts *Lyophyllum* sp. strain Karsten and *Trichoderma asperellum* 302^8^ were grown on oat flake agar (OFA, 30 g/L oat flake, 15 g/L agar)^4^ at 28 °C.

### Motility assays

The motility of the wild-type and mutant strains was tested on R2A agar (pH 6.8 or 5.2) with different agar concentrations. For swimming motility, 2.5 g/L agar was used and 5 μL of overnight culture was dropped on the surface of agar. For swarming motility, the concentration of glucose was elevated to 5 g/L and agar was supplied at 6 g/L. Then, colonies were picked up from agar medium and applied to the surface of swarming agar. For tests of twitching motility, 10 g/L agar was used. Colonies were picked up and introduced by puncturing the agar down to the underlying Petri dish. To aid visualization of cells, 500 mg/L 2,3,5-tetraphenyltetrazolium chloride was added to the medium prior to plate pouring.

### Transmission Electron Microscopy

Bacterial cells were carefully recovered from R2A agar and resuspended in 25 μL phosphate-buffered saline (NaCl 8.0 g/L, KCl 0.2 g/L, Na_2_HPO_4_ 1.44 g/L, KH_2_PO_4_ 0.24 g/L, pH 7.2). Then, the cells were deposited onto carbon-coated copper grids and negatively stained with 2% (w/v) uranyl acetate for 1 min. Cells were observed under a Philips CM120 electron microscopy.

### Preparation of soil microcosms

For all experiments, soil from Gieterveen, the Netherlands, was used. The soil was adjusted to pH 6.8 by adding 0.5% of CaCO_3_. Then, it was autoclaved (121 °C, 27 min) three times, with intermittent incubation at room temperature. Soil microcosms were prepared in three-compartment Petri dishes as described in detail in previous papers^4, 19, 50^. In short, one compartment was filled with OFA and the other two with the soil. The OFA compartment received OFA plugs containing fungal mycelial growth. Five (for *Lyophyllum* sp. strain Karsten) or 3 days later (for *Tricholderma asperellum* 302), when fungi grew over the barrier and reached the two soil-filled compartments, about 5 × 10^5^ bacterial cells were introduced in 50 μl water into the soil at the fungal growth fronts. This procedure established an inoculated soil zone of approximately 3 by 45 mm, with no spread of the bacterial inoculant beyond this zone^4^. Three different experimental treatments were set up: (1) *B. terrae* BS001 wild-type strain alone, (2) *B. terrae* BS001 mutant strain alone (*ΔpilN* or *ΔfliF*, respectively), (3) 1:1 mixtures of wild-type and mutant strains. The Petri dishes were sealed with parafilm and incubated horizontally at 28 °C. Then, samples were destructively taken from the soil compartments at the inoculation and the migration sites (i.e. at the hyphal fronts) at days 4, 7 and 14 (following bacterial inoculation) for *L*. sp. strain Karsten or at day 2 for *T. asperellum* 302. The samples were suspended in water, shaken intensely (1 min, 3 times, with 30 s intervals), diluted, and spread on R2A plates for CFU counting. For each experiment, three replicate microcosms were used.

### Analysis of population composition in the mixed inoculation experiments

The proportions of the wild-type strain and mutant strain in the mixed inoculated samples were determined by colony PCR using primer pairs NM1/NP1 and GF1/SR1, respectively. Minimally 32 colonies were picked up for each replicate. The PCR products were checked by agarose gel electrophoresis and the numbers of colonies producing different-sized amplicons, reporting on either mutant or wild-type, were quantified. Using this methodology, the limit of detection of either strain in this experiment was about 0.03% (1/32) of the total. The actual CFU enumerations that underlie these percentages can be found in Tables S3 and S4.

### Direct competition of mutant strain and wild type strain in broth

The mutant and wild-type strains were introduced into M9 broth [supplied with 2 g/L carbon source (glucose or glycerol)] or LB broth at 1:1 ratio. The cultures were incubated at 28 °C, with shaking, and sampled at different time points. At each sampling, aliquots were diluted and spread onto R2A agar. Following plate incubation and colony counting, the population compositions in the mixed inoculation were checked by colony PCR as described above.

### Statistical analysis of the data

All experiments were performed in triplicate at least. Normality of the data was assessed using collective data of statistically similar groups. One-way analysis of variance (ANOVA) was performed for comparison of more than two groups and two-tailed unequal variance t-test was used to compare the difference between two groups. Differences of the means were considered to be significant at P < 0.05.

## Electronic supplementary material

Supplementary information
