# Supplementary material for: Role of flagella and type four pili in the co-migration of Burkholderia terrae BS001 with fungal hyphae through soil
Source: Sci Rep. 2017 Jun 7;7:2997. doi: 10.1038/s41598-017-02959-8 (PMC5462819; doi:10.1038/s41598-017-02959-8)
Supplement: Supplementary file 1 — Supplementary information [file 41598_2017_2959_MOESM1_ESM.pdf]

Supplementary information

Role of flagella and type four pili in the co-migration of *Burkholderia terrae* BS001 with fungal hyphae through soil

Pu Yang, Miaozhi Zhang, Jan Dirk van Elsas

Supplementary Methods

Construction of T4P mutant of *B. terrae* BS001

In order to construct a T4P mutant strain, the *pilN* gene was knocked out via double crossover allelic exchange using suicide vector pSUP202 (chloramphenicol resistance) [1]. Briefly, homologous fragments flanking *pilN* were amplified by PCR using primer pairs NM2/NR1 and NF1/NP2, respectively. The two fragments were then purified from agarose gel. The recovered fragments were then fused by fusion PCR using primer pairs NM2/NP2 and subsequently ligated into the pGEM-T vector (Promega Corporation, Madison, USA). Following this, the cloned construct was double-digested by HindIII/BamHI and the corresponding fragment was ligated with HindIII/BamHI-digested pSUP202 to make pSUP202-ΔpilN. Next, the pSUP202-ΔpilN construct was transferred to the mobilizing strain *E. coli* S17-1, and then introduced into *B. terrae* BS001 by conjugation. A presumed single cross-over mutant was selected on R2A agar with nitrofurantoin (50 mg/L) and chloramphenicol (25 mg/L), and then streaked to purify. After verification by PCR, one positive colony was used as an inoculum for fresh LB broth, grown and transferred every 24 h to new LB broth without antibiotics. After 7 and 14 transfers, the cultures were diluted and spread on R2A agar without antibiotics. Single colonies were picked randomly and checked for the absence of chloramphenicol resistance. Finally, five chloramphenicol-sensitive strains were streaked to purify and then examined by primer pairs NM1/NP1, NM2/NP2, in order to check for the loss of a 0.5-kb fragment. The desired double-crossover mutant strain was stored at -80 °C in 20% glycerol. All primers are shown in figure S1 and listed in table S5.

Construction of flagellar mutant strain of *B. terrae* BS001

The strategy used in this experiment was basically the same as the one used to construct the T4P mutant strain, with a few modifications (figure S2), as *FliF* was knocked out. Briefly, primer pairs FR1/GF2, FF1/SR2 were used to obtain the homologous fragments flanking *fliF*, and GF2/SR2 were used in the fusion PCR. Then, the relevant fragment was ligated and placed in pGEM-T. Since we failed in ligation after digestion with EcoRI (digestion site designed in the primers, see Table S3), we double-digested the vector with HindIII/BamHI (digestion site was close to the end of the PCR fragment, Supplementary Fig. S8) and subsequently ligated with double-digested pSUP202. The corresponding construct was introduced into *B. terrae* BS001 by conjugation, and a single cross-over strain was selected and verified. The desired mutant strain was then obtained - similar to the above - after serial transfers in fresh LB broth. A set of putative mutants was purified, identified by primer pairs GF2/SR2, GF1/SR1, and a single confirmed one stored at -80 °C in 20% glycerol. All primers are shown in figure S2 and listed in table S5.

Expression of flagellar genes in BS001 wild-type and *ΔfliF* mutant

Overnight cultures of BS001 wild-type and *ΔfliF* mutant were washed and 5 x 10^6 cells of each were introduced in a single spot on swimming agar, respectively. Plates were incubated overnight at room temperature and RNA was then extracted from all growth at the inoculation site using QIAGEN RNeasy Mini kit. The extraction was treated by DNase and applied to SuperScript III First-Strand Synthesis System (Invitrogen) for cDNA synthesis. qPCR were then performed in an Applied Biosystems 7300 Real-Time PCR System. Primers used in qPCR are listed in Table S6.

Reference

1 Simon, R., Priefer, U. & Puhler, A. A broad host range mobilization system for in vivo genetic engineering: transposon mutagenesis in gram negative bacteria. *Nat. Biotech.* **1**, 784-791 (1983).

Figure S1 Overview of *B. terrae* BS001 *pilN* knock-out construction. (a) Primer design for homologous fragments amplification. (b) PCR verification of *pilN* gene knock out. M, GeneRuler 1 kb DNA Ladder (ThermoFisher Scientific). 1, 2, used primer pair NM1/NP1; 3, 4, used primer pair NM2/NP2; 1, 3, used genomic DNA of *ΔpilN* mutant strain as template; 2, 4, used genomic DNA of wild-type strain as template. The PCR products of *ΔpilN* mutant strain were 0.5 kb smaller than wild-type strain.

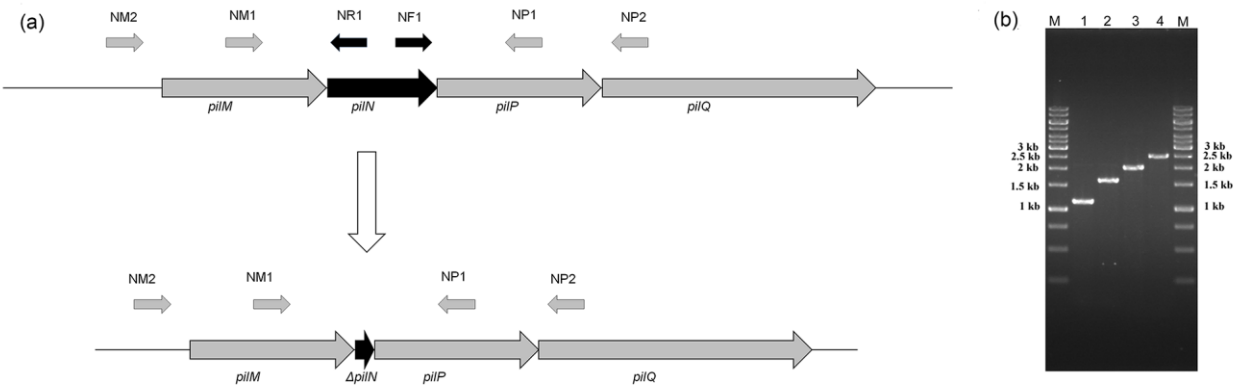

Figure S2 Overview of *B. terrae* BS001 Δ*fliF* knock-out construction. (a) Primer design for homologous fragments amplification. (b) PCR verification of *ΔfliF* gene knock out. M, GeneRuler 1 kb DNA Ladder (ThermoFisher Scientific). 1, 2, used primer pair GF1/SR1; 3, 4, used primer pair GF2/SR2; 1, 3, used genomic DNA of *ΔfliF* mutant strain as template; 2, 4, used genomic DNA of wild-type strain as template. The PCR products of *ΔfliF* mutant strain were 1.7 kb smaller than wild-type strain.

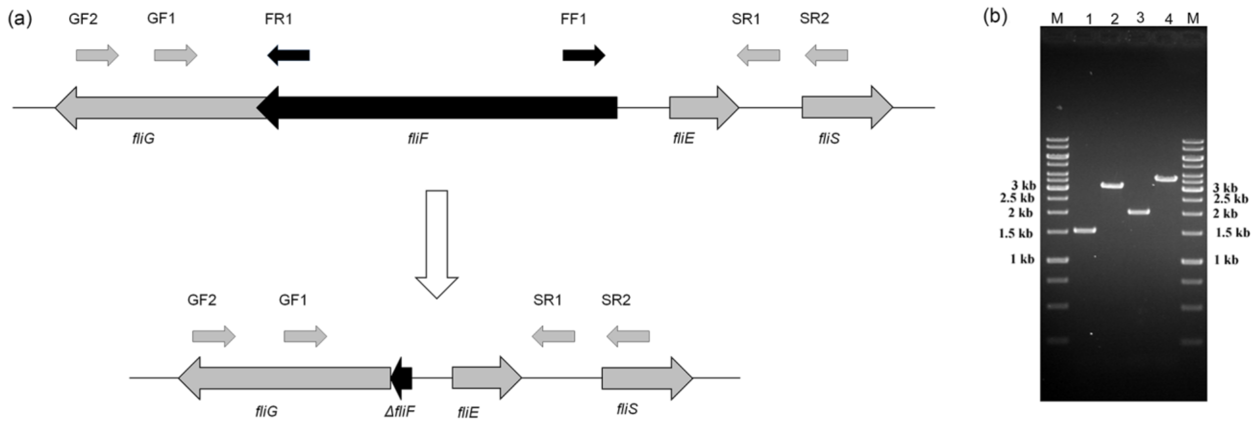

Figure S3 Expression of selected flagellar genes (*fliC*, *fliP*, *fliM*, *fliI* and *fliF*) in BS001 wild-type and BS001 *ΔfliF* mutant using the glyceraldehyde-3-phosphate dehydrogenase gene as the reference gene. The differences between the expression levels of wild-type and mutant were not significant (t-test, p=0.774 for *fliC*, p=0.475 for *fliP*, p= 0.0905 for *fliM*, p=0.209 for *fliI*). Expression of *fliF* was not detected in the *ΔfliF* mutant.

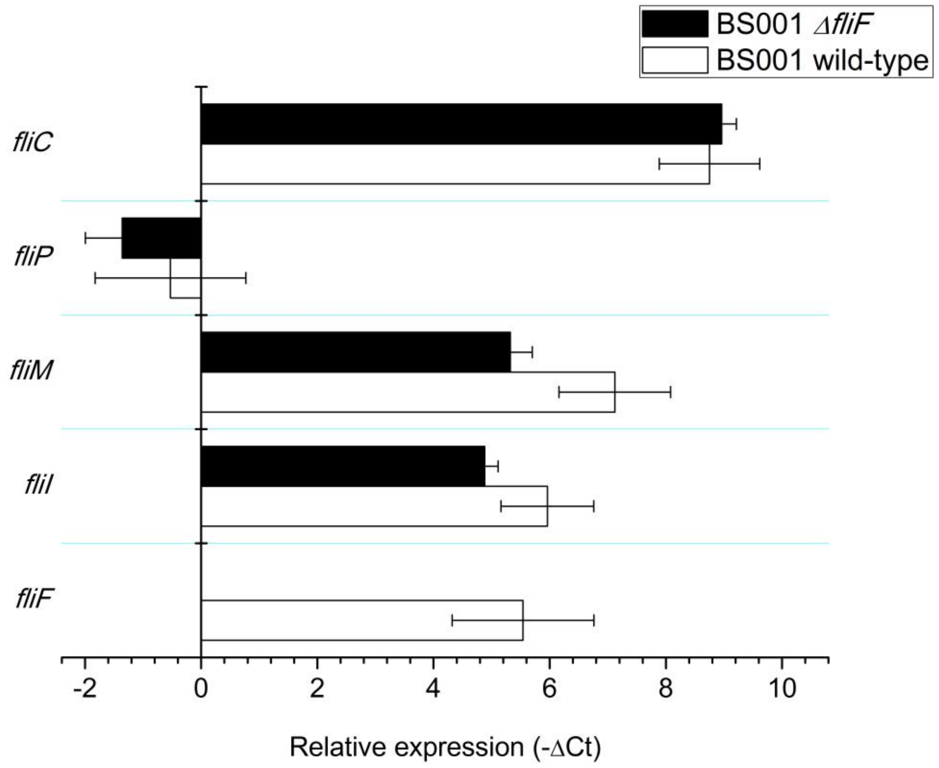

Figure S4 Direct competition between wild-type strain and mutant strain in medium. (a), (b), (c), wild-type and *ΔpilN* mutant strain co-inoculation; (d), (e), (f), wild-type and *ΔfliF* mutant strain co-inoculation; (a), (d), LB broth; (b), (e), M9 medium supplemented with 2 g/L glucose; (c), (f), M9 medium supplemented with 2 g/L glycerol.

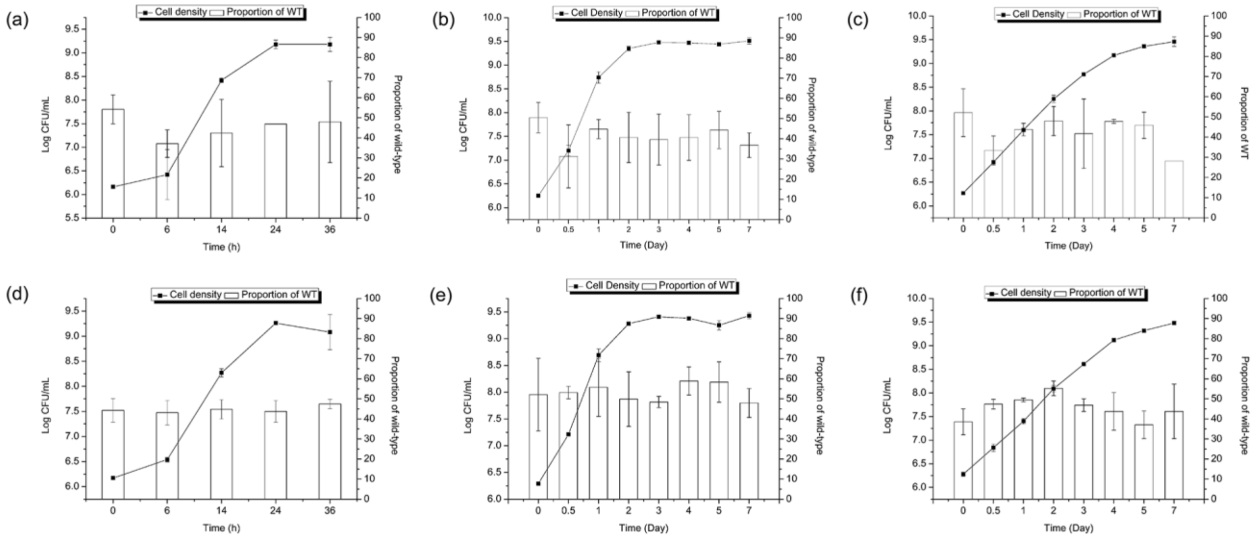

Figure S5 Growth and migration overtime of BS001 strains (wild-type versus mutants) on swimming agar plates. (a) wild-type/*ΔpilN* mutant mixture, diameter development and proportion of mutant strain; (b) wild-type/*ΔpilN* mutant mixture, CFU counts; (c) wild-type/*ΔfliF* mutant mixture, diameter development and proportion of mutant strain; (d) wild-type/*fliF* mutant mixture, CFU counts.

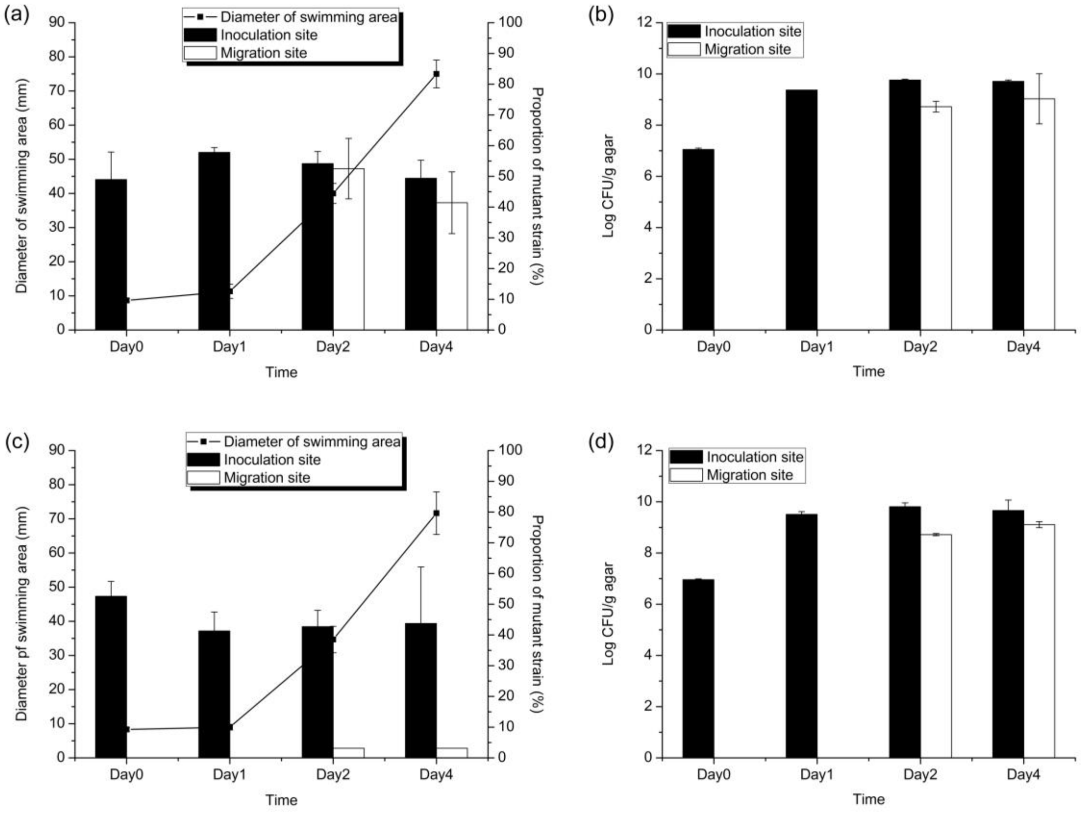

Figure S6 Electron microscopic image of *B. terrae* BS001 wild-type strain recovered from swimming agar. A lot of free flagella we observed in the field. The other strains (*ΔsctD* and *ΔpilN* mutant strains) recovered from swimming agar and swarming agar showed similar results (not shown).

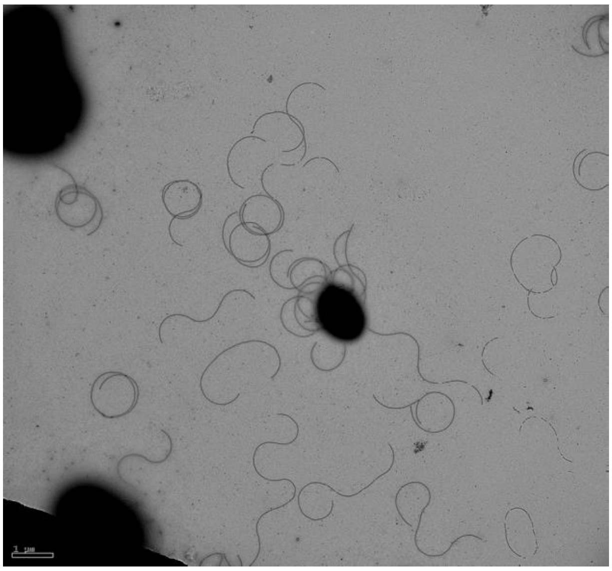

Figure S7 Twitching motility of *Pseudomonas aeruginosa* PA1.

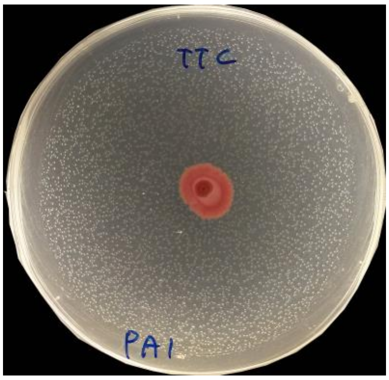

Figure S8 Restriction enzyme digestion site on the homologous fragment for *fliF* knock-out.

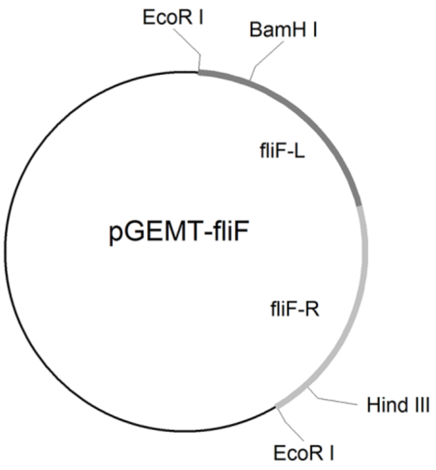

Table S1 Predicted ORFs of flagellum structural proteins and chemotaxis-related proteins in *B. terrae* BS001

Proteins	Gene ID	Gene position		Protein length (aa)	Identity to *Burkholderia multivorans* ATCC BAA-247 (BLASTP)	Predicted function
		Start	End			
Contig00011						
FliC	WQE_01220	137363	138181	272	45%	Flagellin, filament
Contig00012						
FlhF	WQE_01757	331	2172	613	59%	GTPase
FlhG	WQE_01762	2165	3034	289	60%	FlhF inhibitory ATPase
FliA	WQE_01767	3057	3791	244	90%	Transcriptional regulator, sigma factor 70
FlgN	WQE_01772	4735	5181	148	60%	Export chaperone for FlgK and FlgL
FlgM	WQE_01777	5280	5621	113	47%	Anti-sigma-28 factor
FlgA	WQE_01782	5727	6986	419	74%	Basal body, P-ring
FlgB	WQE_01787	7229	7720	163	72%	Basal body, rod
FlgC	WQE_01792	7830	8255	141	89%	Basal body, rod
FlgD	WQE_01797	8267	8980	237	63%	Hook cap
FlgE	WQE_01802	9014	10522	502	64%	Hook
FlgF	WQE_01807	10545	11303	252	75%	Basal body, rod
FlgG	WQE_01812	11355	12143	262	90%	Basal body, rod

FlgH	WQE_01817	12194	12904	236	81%	Basal body, L-ring
FlgI	WQE_01822	12907	14094	395	81%	Basal body, P-ring
FlgJ	WQE_01827	14107	15039	310	66%	Muramidase
YcgR	WQE_01832	15461	16210	249	69%	Regulator, flagellar brake
FlgK	WQE_01837	17220	19193	657	64%	Hook-filament junction
FlgL	WQE_01842	19208	20428	406	56%	Hook
FliR	WQE_01847	21361	22143	260	67%	Exporter
FliQ	WQE_01852	22175	22444	89	69%	Exporter
FliP	WQE_01857	22467	23237	256	89%	Exporter
FliO	WQE_01862	23324	23902	192	64%	Expoter
FliN	WQE_01867	23941	24405	154	87%	Motor switch, C-ring
FliM	WQE_01872	24398	25396	332	90%	Motor switch, C-ring
FliL	WQE_01877	25462	25965	167	63%	Basal body
FliK	WQE_01882	26558	28072	504	65%	Hook length control
FliJ	WQE_01887	28138	28587	149	59%	ATPase ring complex
FliI	WQE_01892	28606	30261	551	76%	ATPase
FliH	WQE_01897	30255	30929	224	65%	ATPase ring complex
FliG	WQE_01902	30922	31917	331	85%	Motor switch, C-ring
FliF	WQE_01907	31907	33688	593	70%	MS-ring
FliE	WQE_01912	33921	34256	111	86%	Hook-basal body

						junction
FliS	WQE_01917	34657	35091	144	78%	Unknown
FliT	WQE_01922	35088	35417	109	56%	Export chaperone?
FliK	WQE_01927	35486	36883	465	60%	Hook length control
FlhB	WQE_01932	36880	37215	111	72%	Exporter
Contig00091						
CheD	WQE_13876	387	1187	266	75%	Chemotaxis, receptor
CheR	WQE_13881	1184	2125	313	81%	Chemotaxis, methyltransferase
MCP	WQE_13886	2371	4158	595	67%	Chemotaxis, Methyl-accepting protein
CheW	WQE_13891	4222	4749	175	89%	Chemotaxis, signal transduction
CheA	WQE_13896	4795	7083	762	92%	Chemotaxis, histidine protein kinase
CheY	WQE_13901	7157	7519	120	69%	Chemotaxis, signal receiver
MotB	WQE_13906	7590	8708	372	75%	Flagellar motor
MotA	WQE_13911	8721	9581	286	94%	Flagellar motor
FlhC	WQE_13916	9741	10352	203	85%	Transcriptional activator
FlhD	WQE_13921	10484	10804	106	84%	Transcriptional activator
FliC	WQE_13976	20288	21106	272	51%	Flagellin, filament

FliD	WQE_13981	21270	22703	477	46%	Flagellar capping protein
Contig00104						
MotA	WQE_16754	34279	35148	289	56%	Flagellar motor
MotB	WQE_16759	35172	36155	327	51%	Flagellar motor
Contig00105						
FliC	WQE_17719	18340	19158	272	47%	Flagellin, filament
Contig00317						
MotA	WQE_43069	2650	3414	254	26%	Flagellar motor
MotB	WQE_43074	3477	4172	231	33%	Flagellar motor

Table S2 Predicted ORFs of type-4 pili related proteins in *B. terrae* BS001

Proteins	Gene ID	Gene position		Protein length (aa)	Identity (organism, GenBank accession no.)[a]	Predicted function
		Start	End			
Contig00130						
PilM	WQE_23373	7121	8056	311	92% (*Burkholderia caribensis* MBA4, ALL63830)	Pilus assembly platform
PilN	WQE_23378	8053	8664	203	90% (*Burkholderia caribensis* MBA4, ALL63831)	Pilus assembly platform
PilO[b]	WQE_23383	8661	9575	304	93% (*Burkholderia caribensis* MBA4, ALL63832)	Pilus assembly platform
PilQ	WQE_23388	9572	11155	527	95% (*Burkholderia caribensis* MBA4, ALL63833)	Secretion pore
Contig00250						
PilA	WQE_36692	1177	1794	205	97% (*Burkholderia caribensis* MBA4, ALL62994)	Major pilin
Contig00372						
PilD	WQE_47879	36158	37087	309	98% (*Burkholderia caribensis* MBA4, ALL63994)	Prepilin peptidase
PilC		37118	38341	407	95% (*Burkholderia caribensis* MBA4, ALL63995)	Inner membrane
PilB		38337	39590	417	91% (*Burkholderia caribensis* MBA4, ALL63996)	ATPase
Contig00037						

PilT	WQE_04472	316	705	129	75% (*Burkholderia multivorans* ATCC BAA-247, AJY19449)	ATPase
PilF	WQE_05897	11349	12326	325	85% (*Burkholderia multivorans* ATCC 17616, BAG47559)	ATPase
Contig00054						
PilT	WQE_06262	60245	60646	133	85% (*Burkholderia multivorans* ATCC BAA-247, AJY18243)	ATPase
Contig00100						
PilF	WQE_16084	9953	10948	331	97% (*Burkholderia fungorum* ATCC BAA-463, AJZ56847)	ATPase
Contig00122						
FimT	WQE_22531	2455	2934	159	79% (*Burkholderia caribensis* MBA4, ALL64227)	Prepilin leader protein

a, identity in protein level (BLASTP)

b, annotated as PilP in *Burkholderia caribensis* MBA4, it also contains PilO domain, COG3167

Table S3 Counts of wild-type and mutant CFUs (per gram of dry soil) in soil microcosms that received wild-type/*ΔpilN* mutant strain mixes (1:1)

Fungal host	Sampling time	Position	Replicates	Number of cells ($X 10^8$ CFU/g dry soil)	
				Wild-type	mutant
Lyophyllum sp. strain Karsten	Day 0	Inoculation site	1	$1.76 X 10^{-3}$	$2.58 X 10^{-3}$
			2	$2.58 X 10^{-3}$	$1.76 X 10^{-3}$
			3	$1.90 X 10^{-3}$	$2.44 X 10^{-3}$
	Day 4	Inoculation site	1	2.61	5.74
			2	1.16	3.98
			3	2.50	10.02
		Migration site	1	5.87	4.02
			2	9.19	2.12
			3	3.01	1.80
	Day 7	Inoculation site	1	1.87	2.58
			2	1.88	3.60
			3	0.70	1.33
		Migration site	1	10.12	4.14
			2	11.40	3.19
			3	12.77	4.44
	Day 14	Inoculation site	1	1.63	3.10
			2	0.57	2.35
			3	1.63	3.10
		Migration site	1	7.63	2.99
			2	12.14	1.73
			3	10.33	5.41
Trichoderma asperellum 302	Day 0	Inoculation site	1	$1.06 X 10^{-3}$	$0.94 X 10^{-3}$
			2	$0.57 X 10^{-3}$	$1.44 X 10^{-3}$
			3	$0.88 X 10^{-3}$	$1.13 X 10^{-3}$
	Day 2	Inoculation site	1	0.50	0.64
			2	0.37	0.37
			3	0.31	0.51
		Migration site	1	1.24	0.85
			2	1.00	0.46
			3	0.72	0.17

Table S4 Counts of wild-type and mutant CFUs (per gram of dry soil) in soil microcosms that received wild-type/*ΔfliF* mutant strain mixes (1:1)

Fungal host	Sampling time	Position	Replicates	Number of cells ($X\ 10^8$ CFU/g dry soil)	
				Wild type	Mutant
Lyophyllum sp. strain Karsten	Day 0	Inoculation site	1	$1.78\ X\ 10^{-3}$	$2.61\ X\ 10^{-3}$
			2	$2.33\ X\ 10^{-3}$	$2.06\ X\ 10^{-3}$
			3	$1.51\ X\ 10^{-3}$	$2.88\ X\ 10^{-3}$
	Day 4	Inoculation site	1	1.79	3.43
			2	1.79	2.62
			3	1.60	1.60
		Migration site	1	8.67	0
			2	13.75	0
			3	13.52	0
	Day 7	Inoculation site	1	1.02	2.23
			2	0.33	4.99
			3	0.64	2.79
		Migration site	1	12.10	0
			2	11.88	0
			3	7.10	0
	Day 14	Inoculation site	1	0.61	2.19
			2	0.84	2.89
			3	0.58	2.05
		Migration site	1	11.19	0
			2	13.28	0
			3	12.58	0
Trichoderma asperellum 302	Day 0	Inoculation site	1	$0.94\ X\ 10^{-3}$	$1.06\ X\ 10^{-3}$
			2	$1.06\ X\ 10^{-3}$	$0.94\ X\ 10^{-3}$
			3	$0.75\ X\ 10^{-3}$	$1.25\ X\ 10^{-3}$
	Day 2	Inoculation site	1	1.25	1.25
			2	2.37	0.45
			3	2.29	0.89
		Migration site	1	7.04	0
			2	6.01	0
			3	6.05	0

Table S5 Primers used in mutant construction

Primer names	Sequence (5'-3')	Restriction enzyme
NM1	GCTAAGCTTCCGCATCTTCGCACGGTGTC	HindIII
NM2	GCTAAGCTTGTTGGTATCTGAGGGAGGAAGGAAT	HindIII
NP1	GCTGGATCCCTCGCAAGCCCTTCCCAAAA	BamHI
NP2	GCTGGATCCGCGTATGGCGTTGAGCCGTAT	BamHI
NF1	**ATCTGTTGCC**ATCAAAGCACACCTGAAATGGGAC	
NR1	**TTGAT**GGCAACAGATTGAAACCGTGGG	
GF1	CGATGACCGACTCTTCGTGATTGC	
GF2	GCGGAATTCCTATCACGATTGCTCCCGACTC	EcoRI
SR1	GCTGGAAGGAAGCGTCAGGAAAGATA	
SR2	GCGGAATTCGGCATTGGCTCCAGAGTGTCCC	EcoRI
FF1	**TCACGACGGT**GCGGGCATCGGGGTTGAT	
FR1	**GCCCGC**ACCGTCGTGAAGAGCTGGGTGTCC	

The restriction sites at the primers are shown by underling. The nucleotides in bold represents overlap sequences used for fusion PCR.

Table S6 Primers used in qPCR

Primer names	Sequences (5'-3')	Detect genes
C297	CGCAAACGGTTACGTCCAGA	*fliC*
C398	ATCAGCTGTTTCGCGGTGTTG	
P165	TGAACACGGTGAAGCCAATCTGA	*fliP*
P376	GATACAGATGGACGAAGCAGTGAA	
M9	ATCCCGCTTCCTTCATCTGTTCC	*fliM*
M149	CCATCAACATTCCCGAGCACAT	
I113	ATGCGGAACGCCTGAAGGACC	*fliI*
I293	GCCTGCTGGTTATTGATCGAGATGC	
F803	TGCTGCGGGTCGTTGTTC	*fliF*
F921	GCGATCCTTGGACCGATGTT	
DHF	ACCTTCTTCGCACCAGCCTT	glyceraldehyde-3-phosphate dehydrogenase gene
DHR	GACCGCTATCAAGAATCCTGCTG	
